# A Rare Case of Isolated Cardiac Sarcoidosis With Negative Biopsy: Diagnosis by Clinical Criteria

**DOI:** 10.7759/cureus.52088

**Published:** 2024-01-11

**Authors:** Tiffany Le, John Dayco, Karthik Ramaseshan, Mariam Zunnu Rain, Luis C Afonso

**Affiliations:** 1 Internal Medicine, Wayne State University Detroit Medical Center, Detroit, USA; 2 Internal Medicine, Detroit Medical Center/Wayne State University School of Medicine, Detroit, USA; 3 Cardiology, Wayne State University Detroit Medical Center, Detroit, USA

**Keywords:** cardiology, chest pain, myocarditis, cardiac sarcoidosis, isolated cardiac sarcoidosis

## Abstract

Cardiac sarcoidosis (CS) is a potentially life-threatening condition that can cause sudden, fatal conduction abnormalities, arrhythmias, and heart failure. The diagnosis of CS is challenging due to nonspecific symptoms and an unclear diagnostic criterion. Although biopsy is the gold standard method, the sensitivity of biopsy is low. About a portion of CS cases are detected through imaging. A unique aspect of our case is that our definitive diagnosis was made based on clinical and imaging criteria alone despite a negative biopsy. Our diagnosis was confirmed further on follow-up with improvement in cardiac function on imaging after a treatment course with corticosteroids. This case highlights the need to have a broad differential and more awareness of this rare etiology and the value of clinical criteria to make a definitive diagnosis.

## Introduction

Sarcoidosis is a systemic disease characterized by noncaseating granulomas that may affect any organ of the body, including the heart. Cardiac sarcoidosis (CS) is reported in only around 5% of those with systemic sarcoidosis [1]. However, autopsy studies and cardiac magnetic resonance imaging (CMR) have detected asymptomatic cardiac involvement in up to 26% of patients with extracardiac sarcoidosis [2]. Given the severity of CS, early diagnosis and prompt treatment are necessary. The diagnosis of CS is challenging due to nonspecific symptoms and an unclear diagnostic criterion previously based on a prior diagnosis of extracardiac sarcoidosis. Although biopsy is the gold standard method, the sensitivity of biopsy is 25-30% [1]. About 35% of CS cases are detected through imaging [1]. Our case demonstrates the challenges of diagnosing CS without endomyocardial biopsy and absent evidence of extracardiac sarcoidosis (i.e. isolated cardiac sarcoidosis).

## Case presentation

A man in his 40s was re-admitted to our hospital with symptoms of chest pain worsened with positional changes, palpitations, diaphoresis, and dyspnea. He reported a variety of diagnoses but felt only partial improvement before the symptoms improved. He had been lost to follow-up on numerous occasions, and his symptoms became progressively worse with each recurrent episode. Over the course of some of these admissions, he underwent extensive cardiac workup with electrocardiogram (EKG), echocardiogram, left heart cardiac catheterization, CMR, and fluorodeoxyglucose positron emission tomography (FDG-PET), ultimately receiving the diagnosis of viral myocarditis. 

**Figure 1:**
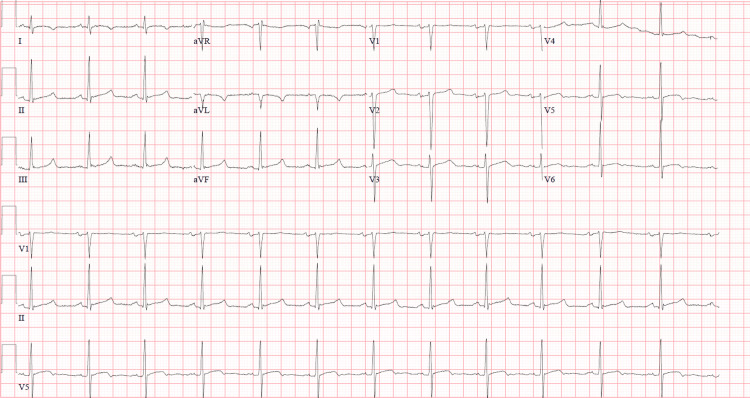
EKG showing normal sinus rhythm

His EKG showed normal sinus rhythm without any atrioventricular blocks (AV), ventricular tachycardias, and ST-segment elevations (Figure 1). Troponin levels were moderately elevated. C-reactive protein and erythrocyte sedimentation rates were normal. Transthoracic echocardiogram (TTE) showed focal mid-anterior and anterior-lateral wall thinning and hypokinesis. An FDG-PET scan showed myocardial glucose uptake along a small area of the mid-anterolateral myocardial wall representing increased metabolic activity and focal myocardial inflammation which was suspected to represent focal viral myocarditis due to a viral URI prior to admission. He was treated with colchicine and indomethacin but was lost to follow-up.

**Figure 2:**
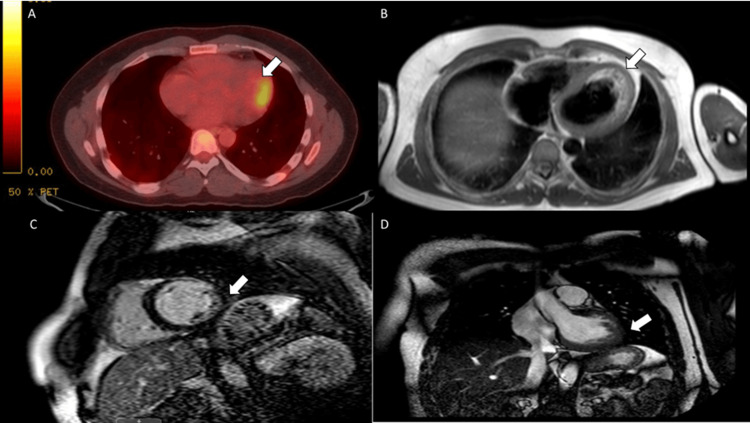
F-18 FDG metabolic imaging A: A small area of the mid-anterolateral myocardial wall increased metabolic activity suggesting focal myocardial inflammation. B: T2 sequences demonstrate focal hyperintense signal involving the mid-anterolateral wall. C: Short-axis view showing left ventricular delayed enhancement consistent with scarring/fibrosis involving the transmural mid-anterolateral wall and subendocardial and mid-wall distal anterolateral and mid and distal anterior wall segment. D: Long-axis view. FDG: Fluorodeoxyglucose

He returned several months later with a similar presentation. This time, he was treated with prednisone for three months and scheduled for outpatient CMR. He missed his appointment and required readmission six months later. The CMR performed during that admission illustrated focal T2 hyperenhancement and delayed hyperenhancement consistent with transmural scarring/fibrosis involving the mid-anterolateral wall and subendocardial fibrosis along to mid to distal anterior wall segments involving the transmural (100% thickness), mid-anterolateral wall, and subendocardial and mid-wall (50% wall thickness) (Figures 2A-2D). Liver biopsy was negative for granuloma.

**Figure 3:**
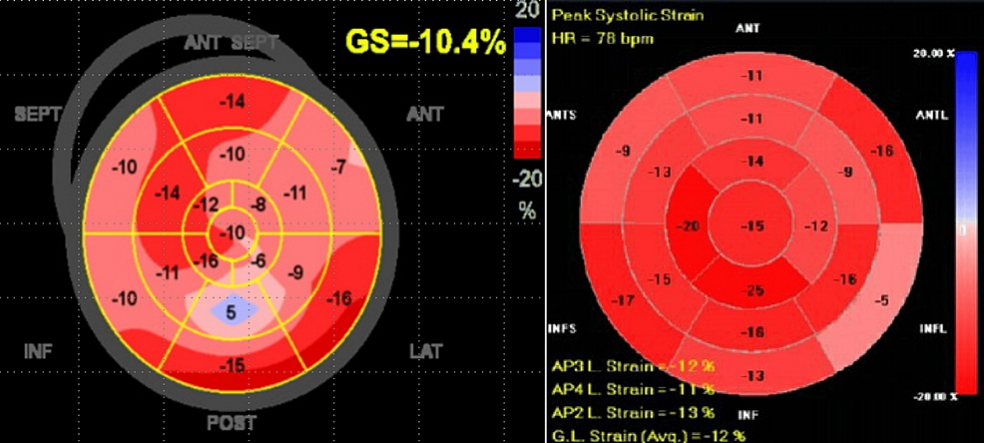
Bullseye plot A: Mid-inferolateral wall with significantly reduced strain displayed in blue and pink regions. B: Longitudinal strain bullseye plot with complete resolution of the inferior lateral wall after treatment, especially when compared to the strain mapping noted from two weeks earlier, prior to the initiation of corticosteroids demonstrating an excellent response to steroids. No change to the mid-anterior wall was noted, likely due to transmural involvement resulting in scarring.

During subsequent admissions, infectious workup was negative for coxsackievirus, parvovirus, Lyme, and Epstein-Barr virus. CT thorax did not show evidence of mediastinal or hilar lymphadenopathy. Liver biopsy was negative for granuloma. Based on CMR and PET findings and recurrent symptoms, there was a high suspicion of isolated CS. His later TTE showed a decline in the ejection fraction (EF) to 45 - 50% and a global average longitudinal systolic strain of 10.4%. Hypokinesis and attenuated strain along the mid-inferolateral wall were now evident, along with previously reported mid-anterolateral findings (Figure 3, Video 1). The ophthalmologic exam did not show uveitis. Bronchoalveolar lavage with biopsy did not show granulomas. He was discharged on prednisone for suspected CS due to his decrease in the EF to < 50%, CMR, and PET findings.

**Video 1:** Apical four-chamber view Septal wall hypertrophy, paradoxical basal inferoseptal akinesis, and reduced mid inferior lateral wall strain

Two weeks later, he was in the ambulatory clinic and endorsed the resolution of chest pain, and a follow-up TTE showed a complete resolution of inferolateral wall motion abnormalities but no change in mid-anterior wall hypokinesis (likely focal scarring) consistent with a gratifying response to steroid treatment (Figure 3, Video 2). He was advised to continue the steroid taper and instructed to follow up in the clinic to assess the response to treatment.

**Video 2:** Apical four-chamber view Strain resolution responsive to treatment myocardial inflammation.

## Discussion

There are currently no international guidelines for CS, but there are two proposed diagnosis guidelines [3]. The diagnosis of CS can be challenging. Based on Japanese Circulation Society 2016 guidelines, which integrate evidence of noncaseating granuloma or clinical diagnosis [1], two of the five major criteria or one major and at least two minor criteria are met. Major criteria encompass AV-block, basal thinning of the interventricular septum, positive gallium uptake in the heart, and depressed EF of <50%. Minor criteria include regional abnormal wall motion, PET or CMR delayed enhancement of myocardium, abnormal EKG findings, and endomyocardial biopsy which shows interstitial fibrosis or monocyte infiltration [1]. According to Heart Rhythm Society guidelines, there are two ways to diagnose CS. Histological evidence requires noncaseating granulomas with no other cause [3]. Clinical diagnosis in a noninvasive way requires the histologic diagnosis of extracardiac sarcoidosis and one or more of the following including immunosuppression responsive cardiomyopathy or heart block, unexplained reduced EF <40%, repeated unexplained sustained ventricular tachycardia, patchy uptake of PET, and late gadolinium enhancement on CMR [3]. Other causes of cardiac manifestations must be ruled out. As demonstrated in our case, the diagnosis was initially suspected to be viral myocarditis. In hindsight, the diagnosis was delayed because PET/CMR is nonspecific and myocarditis can mimic CS in imaging studies [2]. Our case did not present with typical findings of hilar lymphadenopathy, erythema nodosum, rash, and uveitis. Multiple attempts of extracardiac biopsies were inconclusive for noncaseating granulomas. Infectious etiology was not likely given the time course over years of recurrent symptoms. The duration of symptoms and recurrent flares appear to be consistent with an empiric diagnosis of isolated CS. The diagnosis was finally made when he met the above criteria when his systolic function decreased to < 50%, PET demonstrated focal uptake, and CMR with delayed enhancement in the myocardium in a noncoronary distribution. Additional supporting evidence should include global longitudinal strain (GLS) on TTE. It could help screen CS and predict adverse events as it can predict subclinical myocardial involvement [1,2,4]. Notwithstanding the challenges involved, our case highlights the importance of recognition of CS, the incorporation of multimodality imaging, and clinical correlation for diagnosis when biopsy is inaccessible. 

Other manifestations of CS include blocks (AV-node and bundle branches), left ventricular aneurysm, basal anteroseptal thinning, regional contractile abnormalities (may involve the right ventricle), systolic dysfunction valvular dysfunction, supraventricular arrhythmia, and ventricular tachycardia with potentially fatal consequences [5]. In a cohort study of patients with CS, those with CS and GLS score-> -17% had worse outcomes with ventricular tachycardia than those without [2]. While steroids remain the mainstay of treatment, failure of steroid therapy after three months should prompt consideration of second-line agents including mycophenolate mofetil, cyclophosphamide, methotrexate, and azathioprine. As a last resort antitumor necrosis factor, biologicals like infliximab and adalimumab are recommended. FDG-PET scans can help monitor response to treatment. Of note, right ventricular FDG uptake generally presents a poor prognosis. 

The presence of sustained or nonsustained VT, unexplained syncope, cardiac arrest, and left ventricular ejection contraction <35% despite immunosuppressive therapy represent indications for an implantable cardiac defibrillator.

## Conclusions

CS, involving only the heart, only makes up a small percentage of all sarcoidosis cases which makes diagnosis of this condition extremely challenging. Diagnosis of CS requires a high index of suspicion and can often mimic other etiology, in particular viral myocarditis, which itself cannot be conclusively ruled out. The recurrent presentation of similar symptoms along with the clinical diagnostic criteria including imaging findings can help make a definitive diagnosis of CS. Management primarily consists of corticosteroids; in refractory cases, other antirheumatics and biologic agents can be considered. 
